# Percutaneous application of allogeneic adipose-derived mesenchymal stem cell in dogs submitted to minimally invasive plate osteosynthesis of the tibia

**DOI:** 10.1590/ACB360206

**Published:** 2021-02-22

**Authors:** Guilherme Galhardo Franco, Bruno Watanabe Minto, Rafael Manzini Dreibi, Jose Sergio Costa, Luis Gustavo Gosuen Gonçalves Dias

**Affiliations:** 1 DVM, MSc, PhD. Universidade Federal do Espírito Santo – Center for Agricultural Sciences and Engineering – Department of Veterinary Medicine – Alegre (ES), Brazil.; 2 DVM, MSc, PhD. Universidade Estadual Paulista – Faculty of Agrarian and Veterinary Sciences – Department of Veterinary Clinic and Surgery – Jaboticabal (SP), Brazil.; 3 DVM, MSc. Universidade Estadual Paulista – Faculty of Agrarian and Veterinary Sciences – Department of Veterinary Clinic and Surgery – Jaboticabal (SP), Brazil.

**Keywords:** AD-MSC, Fracture Healing, MIPO

## Abstract

**Purpose:**

To evaluate clinical outcome following minimally invasive plate osteosynthesis (MIPO) associated with percutaneous transplantation of allogeneic adipose-derived mesenchymal stem cells (AD-MSC) at the tibial fracture site in dogs.

**Methods:**

Thirty-six dogs presenting with nonarticular complete tibial fracture were included in this study. All fractures were treated by the same MIPO technique. The animals were divided in group 1 (n = 20) received a percutaneous application of 3 × 10^6^ AD-MSC at the fracture site and group 2 (n = 16) did not receive any adjuvant treatment. Postoperative radiographic examinations were made at 15, 30, 60, 90 and 120 days.

**Results:**

Fifty-eight percent of the patients were classified as skeletally immature. The median weight of the animals was 18.8 kg. The mean radiographic union time differed statistically between the AD-MSC group (28.5 days) and the control group (70.3 days). Sixty percent of dogs in group 1 and 56.25% of the group 2 were considered immature.

**Conclusions:**

The use of allogeneic AD-MSC cell therapy and MIPO is a safe, viable and effective technique for promoting bone healing in nonarticular tibial fractures in dogs.

## Introduction

Biological fracture treatment requires surgical techniques that ensure maximum preservation of integrity and vascularization of adjacent soft tissues, with appropriate implant selection^1^.

Minimally invasive plate osteosynthesis (MIPO) is the most recent evolution of biological osteosynthesis. In this technique, the fracture site is not exposed, and bone fragments are reduced using indirect techniques^2^^,^ 3. The use of MIPO in the treatment of diaphyseal and metaphyseal tibial fractures in dogs is associated with both faster bone healing compared to traditional techniques and low rates of complications^4^^–^ 6.

The autologous bone graft is still considered the gold standard for reducing healing time, or for use in cases where bone regeneration is impaired. However, in these cases, surgical access to the donor site is required and this may result in complications^7^. Less invasive techniques have been proposed to promote bone regeneration. Mesenchymal stem cell therapy appears to modulate the inflammatory response, decrease fibrous scar tissue around fractures, improve the formation of functional bone and correct insufficient osteogenesis. Stem cells can be isolated from a variety of tissues and may be autologous or allogeneic. These favors bone healing and can be used as complementary therapy in the treatment of complex fractures or after nonunion^8^.

There are few veterinary studies that have evaluated the use of cell therapy in MIPO. This was a prospective study describing the use of MIPO in association with adipose-derived mesenchymal stem cell therapy (AD-MSC) in 20 dogs with diaphyseal and/or metaphyseal tibial fractures.

## Methods

The study was approved by the ethics committee on the use of animals at Universidade Estadual Paulista, Jaboticabal Campus, under protocol 013579/17. This study included 36 dogs with nonarticular, closed tibial fractures, with or without concomitant fibular fractures. The dogs were divided into two experimental groups. Animals in group 1 received treatment with AD-MSC (n = 20) and for group 2 (n = 16) only MIPO was performed. All animals were treated with MIPO, but the dogs in group 1 received percutaneous transplantation of allogeneic AD-MSC at the fracture site.

Animals that presented with obvious signs of malnutrition (low body condition score, dehydration, anorexia, lethargy), or other systemic disease that could compromise bone healing such as renal, cardiac, respiratory, liver and hormonal dysfunctions were excluded from the study. Dogs in which the use of AD-MSC therapy was contraindicated (such as the presence of neoplasia or focus of infection) were also excluded from the study.

### Preoperative planning

The patients underwent radiographic examination of the affected tibia using two orthogonal projections (craniocaudal and mediolateral) to classify the type and site fracture. The intact tibia of the contralateral limb was radiographed to aid preoperative planning evaluation of limb length and guiding the molding of the implant. Bridging plates applied to the medial face of the tibia were used for fracture fixation, respecting the maximum ratio of 50% of the total number of holes in the plate to the total number of screws used. The size of the implants was selected based on the measurements performed on the preoperative radiograph and the size and body mass of the patient. An intramedullary pin was not placed in conjunction with the bone plate in any case

### Obtaining adipose-derived mesenchymalstem cells

A healthy, 1-year-old female donor dog was used for adipose tissue collection. The donor was anesthetized and approximately 25 g of adipose tissue was collected from an incision in the lumbar region. The adipose tissue was placed in a 50 mL tube containing sterile phosphate buffered saline (PBS, Reprodux, Brazil) for 2 min. Immediately after this, it was removed from the first washing tube and placed in the second washing tube for 3 min. Tissue was stored in a tube containing transport medium (PBS) and refrigerated at 4 to 8 °C until transported to the laboratory for cell culture.

### Adipose tissue processing and cell culture

The adipose tissue was washed several times in PBS to remove cellular debris and excess blood. Soon after, it was cut into small particles and placed in contact with the collagenase 0.075% concentration in PBS with low Ca and Mg and hyaluronidase 0.1% (1 mg/mL) solution so that enzymatic digestion could be carried out. This mixture was centrifuged for 15 min at 1250 rotations per minute (RPM) and the cell button was resuspended. The same procedure was repeated four times. The trypan blue exclusion method was performed. This process constituted the preparation of the stromal vascular fraction.

After obtaining the AD-MSC, they were placed in 25 cm^3^ culture bottles containing TCM 199 medium. The culture bottles were placed in cell culture greenhouses at a temperature of 39.5 °C and 5% CO_2_. After approximately 7 days of cultivation, the cell was raised and a new cell culture was started in a 75 cm^3^ bottle. When 80% of cell confluence was obtained, after 5–7 days, trypsinization was performed. The cell culture medium was changed every 2 days. Some cells were retained in the culture bottle and about 1 million cells were conditioned in straws with cellular freezing medium containing 80% Dulbecco’s modified eagle medium (DMEM, Sigma-Aldrich, EUA), 10% dimethyl sulfoxide (DMSO, Sigma-Aldrich, EUA) and 10% fetal bovine serum (SFB, GIBCO, EUA) and kept frozen in liquid nitrogen until use. As a standard procedure for quality control of cell culture, some of the retained cells were induced differentiate into bone tissue. The cells were characterized by flow cytometry with image quantification and identification of molecular markers. Molecular characterization tests for MSCs were performed as determined by the International Society for Cell Therapy. 1 × 10^6^ cells with antibodies were incubated, these being: anti-Human mouse CD29-RD1 (Beckman Counter, USA), anti-Equine mouse CD44-FITC (AbD Serotec, USA), primary CD90 anti-Canine goat (Washington State University, USA) and anti-Goat conjugated mouse IgM AF594 (Thermo Sci., USA) (secondary). And yet the anti-Human rat anti-Human CD34-FITC (Invitrogen, EUA) surface marker. The maintenance of pluripotency of MSCs was analyzed by the presence of two transcription factors (intranuclear markers) SOX2 and OCT3/4. The markers were assessed using the image flow cytometer immunophenotyping method (Amnis, Luminex corporation, United States). The MSCs showed high adhesion capacity to plastic, phenotypic characteristics typical of MSCs and were attached to the bottom of the bottle with a fusiform shape when reaching 80% confluence. The results of the immunophenotyping showed that 90% of the cells expressed the three surface undifferentiation markers CD29, CD44 and CD90 and had low expression of the negative marker CD34. Still, 96% showed expression of pluripotency transcription factors SOX2 and 92% of OCT3/4.

### Thawing for the percutaneous application of AD-MSC at the fracture site

Three straws, containing 1 million AD-MSC each, were thawed in a laboratory set up near the operating room approximately 25 min before the end of the MIPO procedure. First, three straws were removed from liquid nitrogen and held for 10 s at room temperature. Immediately, they were immersed for 30 s in a water bath at 37.5 °C. After complete thawing, the straws were cut, and their contents added to a falcon tube containing 5 mL of thawing PBS medium. This tube was centrifuged for 3 min at 1250 RPM. All supernatant was discarded keeping only the cellular button formed at the bottom of the tube which was resuspended in 5 mL of cellular washing medium with the aid of a pipette whirling the PBS medium. The tube was the centrifuged for 3 min at 1250 RPM and the same process of cell washing was repeated two more times. After the last centrifugation, the supernatant of the washing medium was discarded, leaving only the cellular button, which was resuspended in 1 or 2 mL of 0.9% sterile saline solution, depending on the patient’s size. This solution containing 3 million AD-MSC was stored in a 3 mL syringe coupled to a 30 × 0.8 mm hypodermic needle for percutaneous application at the site of the fracture immediately after the end of MIPO.

The patients were anesthetized according to the standard protocol used in the hospital. Cephalotin (KEFLIN, ABL, Brazil) (22 mg/kg) was administered intravenously 30 min before the incision and, when necessary, every 90 min during the surgical procedure.

**Figure 1:**
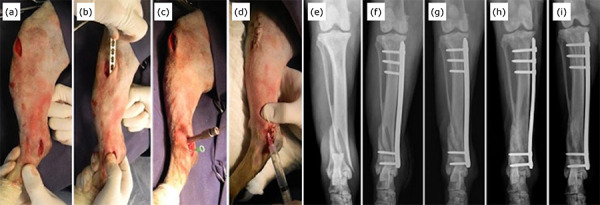
Intraoperative photographs of the minimally invasive osteosynthesis procedure with plate and application of mesenchymal stem cells derived from adipose tissue (**a–d**) and radiographic sequence in the craniocaudal projection (**e–i**) of a dog (dog 20), without defined race (MBD), 15 years of age, with a body mass of 13 kg and distal metaphyseal fracture of tibia in spiral, complete, closed with the fractured fibula. The fracture was indirectly reduced by manual traction (**a**) and the plate was inserted through the proximal access and slipped through the epiperiosteal tunnel over the medial surface of the tibia (**b**) and the bone perforations for application of the screws were performed by distal and proximal accesses. A needle was inserted into the joint space to delimit the tibia (**c**). After dermorrhaphy, 3 million allogeneic AD-MSC diluted in 2 mL of 0.9% saline solution were percutaneously applied to the focus of the fracture using a hypodermic needle and syringe (**d**). The distal metaphyseal spiral tibial fracture (**e**) was treated with a 2.7 mm 12-hole locked plate, applying three proximal locked screws and two distal locked screws **(f**). Radiographic follow-up of the fracture at 15 (**g**), 30 (**h**) and 60 days (**i**), demonstrating bone healing.

The fracture was reduced in a closed and indirect manner, with minimal manipulation (Fig. 1). The surgical approach was performed as described in the literature^5^. The plate was contoured in some patients. The number of screws to be placed was determined according to individual fracture requirements. Blocked plates were used in the systems 1.5, 2.0, 2.4, 2.7, 3.5 and 4.5 mm and all the implants were manufactured by Focus (Focus Veterinary Orthopedics, Brazil). The proximal and distal incisions were then closed in layers using monofilament absorbable sutures.

**Figure 2:**
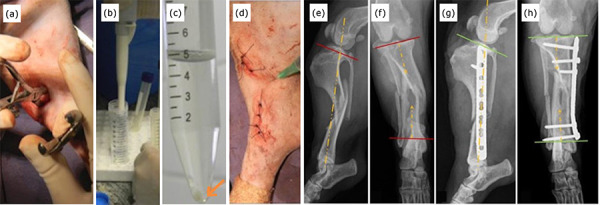
Pictures of the minimally invasive osteosynthesis procedure with plate and application of mesenchymal stem cells derived from adipose tissue (**a–d**) and radiographic images (**e-h**) of a 5-year-old dog without a defined race, body mass of 7.1 kg with spiral diaphyseal fracture of the tibia in the middle third with the fractured fibula. The fracture was indirectly reduced with the aid of bone tweezers applied to the bone through the proximal and distal approaches (**a**). MSC-AD washed in 5 mL of cell wash solution using a pipette (**b**). Photograph showing the cellular button (orange arrow) formed after centrifugation at 1250 RPM for 3 min performed in the process of thawing and cell washing (**c**). After dermorrhaphy, 3 million allogeneic AD-MSC diluted in 2 mL of 0.9% saline solution were percutaneously applied to the focus of the fracture using a hypodermic needle and syringe (**d**). Preoperative radiographic images of the fracture (**e, f**) and in the immediate postoperative period, demonstrating the correction of valgus and recurvatum deviations (**g, h**).

After skin suturing, a 30 × 0.8 mm caliber needle was inserted into the site of the fracture percutaneously by palpation and 1 mL of sterile saline solution containing 3 million mesenchymal stem cells derived from allergenic adipose tissue was injected (Fig. 2).

Antibiotics (cephalexin at 25 mg/kg every 12 h) were prescribed for 10 days, anti-inflammatory drugs (meloxicam at 0.2 mg/kg on the first day and 0.1 mg/kg on the other days every 24 h) for 3 days and tramadol hydrochloride (at 3 mg/kg every 8 h for 5 days) as an analgesic.

### Postoperative evaluation

Radiographic examinations of the affected tibia in two orthogonal projections were performed preoperatively, immediate postoperatively and at 15, 30, 60, 90 and 120 days postoperatively.

Bone healing was evaluated at all radiographic evaluations and clinical union was recorded when there was bone callus bridging the fragments, or > 50% of the tibial diameter at the fracture site in at least 3 of the 4 cortical views in the 2 orthogonal radiographic projections. Bone healing was recorded when cortical and medullary continuity at the fracture site was radiographically observed, as previously described^6^. The radiographs were evaluated blindly and randomized by a single evaluator.

Minor complications were defined as those easily solved with drug therapy not requiring surgical intervention (edema, hematoma, seroma, suture dehiscence). Major complications were defined as those that required more severe revision surgery under general anesthesia (implant failure, severe failure in alignment, osteomyelitis, screw inserted into the joint space).

### Statistical analysis

Statistical analysis was performed using the software Prism GraphPad for Windows. Age, weight and time until bone union data were represented by median ± interquartile range and compared by unpaired Wilcoxon rank sum test. The means and standard deviations of the healing time of the fracture of the control group and mesenchymal stem cell were compared. For all analyses statistically significance was set at p < 0.05.

## Results

Thirty-six dogs were included in the study. There were equal numbers of males and females. The median age at the time of trauma was 42.0 months (3–180 months) (Table 1). Fifty-eight percent of the patients were classified as skeletally immature, that is, growth plates were open on preoperative radiographs. Additionally, 8.3% of the patients were older than 13 years at the time of trauma. The median weight of the animals was 18.8 kg (14.5–36 kg). The age and weight of the animals did not differ statistically between groups.

**Table 1:** Summary of the clinical data of the dogs in both groups included in this study with tibial fractures treated with MIPO and in the G2 percutaneous injection of the allogeneic AD-MSC.

Dogs		Age (months)		Breed		Weight (kg)		Fracture		Implants		Bone union (days)
G1-1		3		Dober.		5.6		Diaf., trans., middle		2.0 mm, 8 H., 4 screws		15
G1-2		3		MBD		24.2		Diaf., obliq., distal		3.5 mm, 11 H., 4 screws		30
G1-3		5		Boxer		10		Diaf., obliq., middle		2.0 mm, 10 H., 4 screws		15
G1-4		7		MBD		6.3		Diaf., obliq., middle		2.0 mm, 10 H., 4 screws		15
G1-5		7		MBD		7.1		Diaf., obliq., middle		2.0 mm, 9 H., 4 screws		15
G1-6		8		Shih Tzu		6.6		Diaf., spiral, middle		2.0 mm, 8 H., 4 screws		30
G1-7		9		MBD		7.9		Diaf., trans., middle		2.0 mm, 10 H., 4 screws		60
G1-8		9		MBD		20.5		Diaf., spiral, middle		3.5 mm, 11 H., 4 screws		15
G1-9		9		Eng. Bull.		16		Diaf., spiral, middle		2.7 mm, 9 H., 5 screws		30
G1-10		11		Am.Staf.Ter.		27		Diaf., obliq., prox.		3.5 mm, 10 H., 4 screws		30
G1-11		12		Rott.		25.8		Diaf., obliq., middle		2.7 mm, 12 H., 5 screws		15
G1-12		12		Bull Ter.		14		Diaf., obliq., middle		2.7 mm, 11 H., 5 screws		30
G1-13		24		MBD		17		Diaf., spiral, middle		2.7 mm, 8 H., 4 screws		30
G1-14		24		MBD		23.2		Meta., com., prox.		3.5 mm, 12 H., 5 screws		30
G1-15		48		C.Chow		25.2		Diaf., com., middle		3.5 mm, 11 H., 5 screws		30
G1-16		60		MBD		7.1		Diaf., spiral, middle		2.0 mm, 9 H., 5 screws.		30
G1-17		96		MBD		18		Diaf., com., middle		2.7 mm, 12 H., 5 screws		30
G1-18		156		Rott.		36		Meta., com., distal		3.5 mm B, 14H., 6 screws		30
G1-19		156		Pinscher		2.7		Diaf., obliq., prox.		1.5 mm, 10 H., 4 screws		60
G1-20		180		MBD		13		Meta., spiral., distal		2.7 mm, 12 H., 5 screws		30
G2-1		4		Poodle		5.2		Diaf., trans., middle		2.0 mm, 10H., 3 screws		60
G2-2		7		MBD		10		Diaf., trans., middle		2.7 mm, 7H., 2 screws		90
G2-3		8		Border Collie		25		Diaf., obliq., middle		3.5 mm, 10H., 2screws		60
G2-4		8		Labrador Retriever		20		Diaf., obliq., middle		3.5 mm, 11H., 2 screws		15
G2-5		8		Labrador Retriever		30		Diaf., trans., prox.		3.5 mm, 11H., 3 screws		60
G2-6		9		MBD		16		Diaf., com., middle		2.7 mm, 10H., 3 screws		30
G2-7		11		Blue Heeler		15		Diaf., trans., middle		2.7 mm, 10H., 3 screws		60
G2-8		12		MBD		20		Diaf. com., middle		2.7 mm, 9H., 2 screws		30
G2-9		12		MBD		30		Diaf., trans., middle		3.5 mm, 11H., 3 screws		60
G2-10		48		MBD		8		Diaf., com., middle		2.7 mm, 7H., 2 screws		90
G2-11		60		MBD		23		Diaf., com., middle		3.5 mm, 12H., 2 screws		120
G2-12		60		MBD		20		Diaf., trans., middle		4.5 mm, 12H., 3 screws		90
G2-13		60		MBD		5		Diaf., trans., prox.		2.0 mm, 10H., 3 screws		90
G2-14		72		Dachshund		7		Diaf., trans., middle		2.0 mm, 7H., 2 screws		120
G2-15		108		Dachshund		9		Diaf., trans., middle		2.4 mm, 7H., 2 screws		60
G2-16		120		MBD		5.5		Diaf., trans., distal		2.0 mm, 9H., 2 screws		90

G1. Group 1 – mesenchymal stem cell group; G2. Group 2 – Control group; y., years; m., months; MBD, mixed breed dog; Rott., Rottweiler; Bull Ter., Bull Terrier; Dober., Dobermann Pinscher.; Am.Staf.Ter., American Staffordshire Terrier; C.Chow, Chow-Chow; Eng. Bull., English Bulldog; diaf., diaphyseal; meta., metaphyseal; obliq., oblique; trans., transverse; com., comminuted; B., Broad; H., holes; kg, kilogram.

All fractures were complete and closed. Of the tibial fractures, 33 (91.7%) were diaphyseal and 3 (8.3%) metaphyseal, (27 in middle third, 5 in proximal third and 4 in distal third). Forty-five point four percent of the fractures were oblique or spiral, 33.3% were transverse and 22.2% were comminuted. All surgeries were performed by the same surgical team, and the median time between trauma and the surgical procedure was 4 days (3–6 days) (minimum of 1 day and maximum of 15 days). No intraoperative complications were observed.

Different lengths and implant systems were used according to the size of each patient and each tibia. The most commonly used systems were 2.0 (30.5%), 3.5 (30.5%) and 2.7 mm (30.5%). In only one case systems used were 1.5 (2.77%), 2.4 (2.77%) and 4.5 mm (2.77%). In no case was an intramedullary pin used as an additional fixation method or even as an indirect method of fracture reduction.

**Figure 3:**
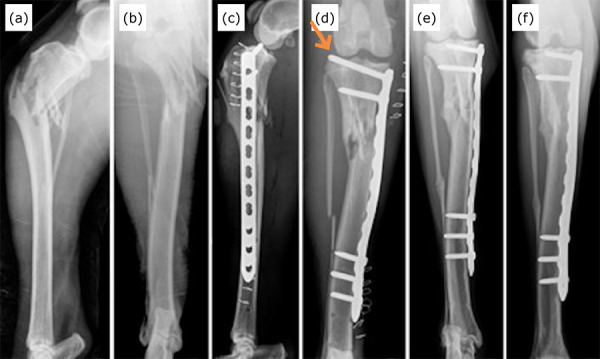
Craniocaudal and mediolateral radiographic images of the tibia of the dog G1-14, without defined race of 2 years of age, with body mass 23.2 kg proximal metaphyseal fracture of the tibia, comminuted and with the fibula fractured in the preoperative period (**a, b**). The immediate postoperative radiographs (**c, d**) demonstrated the complication of screw insertion in the knee joint space (orange arrow). Craniocaudal radiographic projections with 60 days (**e**) and 90 days (**f**) postoperatively, demonstrating good bone healing. The most proximal screw is observed without penetrating the joint space after replacement (**e, f**).

**Figure 4:**
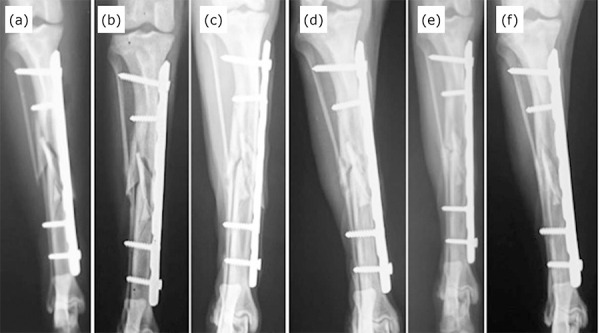
Craniocaudal radiographic images of the tibia of the dog 11 from the control group (mongrel, 5 years of age and 23 kg). Note a mid-diaphyseal comminuted fracture of tibia and fibula in the immediate postoperative period (**a**). Craniocaudal radiographic projections after 15 (**b**), 30 (**c**), 60 (**d**) and 90 days postoperatively (**e**). Note the radiographic clinical union at 120 days postoperatively (**f**).

The mean time of clinical radiographic union differed statistically between groups. The median time of clinical radiographic union was 28.5 days (minimum of 15 days and maximum of 60 days) in group 1 (Fig. 3) and 70.3 days in group 2 (minimum 15 days and maximum of 120 days) (Fig. 4 and Table 2). Sixty percent of dogs in group 1 were considered immature while 43.75% of dogs in group 2 were considered skeletally mature.

**Table 2:** Median ± interquartile range (IQR) of dogs from group 1 (percutaneous application of allogeneic AD-MSC at the fracture site) and group 2 (control).

		Group 1 (n =20)				Group 2 (n =16)				P-value
		Median		IQR		Median		IQR		
Age, months		11.5		43.25		12.0		52.00		0.7015
Weight, kg		15.0		16.35		15.5		13.00		0.9112
Bone union, days		30.0b		15.00		60.0a		30.00		< 0.0001

Median values followed with different superscript letters on the same row represent a significant difference (p < 0.05), as obtained using Wilcoxon rank sum test.

Minor complications included the formation of seroma at the distal access site in one of the cases, which was effectively treated clinically, and the loosening (pullout) of the more proximal screw in two cases, but with no effect on bone healing or clinical healing.

Three major complications were observed (8.33%). Of these, two were the incursion of the most proximal screw end into the joint space of the stifle. A major complication occurred in dog number 3, in which unacceptable apposition was seen in the immediate postoperative radiographs, causing recurvatum deviation and moderate valgus (Fig. 3). No other complications were observed.

## Discussion

The results of this study suggest that the use of MIPO and AD-MSC can accelerate bone regeneration in nonarticular fractures of the tibia. In the postoperative follow-up period, no local or systemic changes compatible with rejection or reaction to cell therapy were noted. Mesenchymal stem cells express molecules from the main Class I histocompatibility complex (MHC I) but have no markers for Class II histocompatibility molecules (MHC II). For these reasons, MSCs are considered nonimmunogenic cells, i.e., incapable of inducing alloreactivity in mammals^9^. This property is extremely important because it prevents signs of rejection after the transplantation of allogeneic MSCs. Admittedly, the cells used in the present study were not examined for MHC I or II expression. However, there was a notable absence of adverse damage when using allogenic AD-MSC, even without immunosuppressive therapies^10^.

Allogeneic MSCs are currently routinely used in veterinary practice since they are available from frozen cell banks in liquid nitrogen. There are several reasons for the choice of AD-MSC in this study. Several *in vitro* and *in vivo* studies have shown promising results in the improvement of bone repair using cell therapy^8^^,^ 11 ^–^ 14. Subcutaneous adipose tissue is a rich source of AD-MSC, available in large quantities, and relatively easy to obtain^15^. In addition, recent reports have demonstrated that, under appropriate conditions, AD-MSC have the potential to differentiate into an osteogenic lineage as efficiently as bone marrow-derived MSCs^16^.

The process of neoangiogenesis is essential for restoring the blood supply of fracture fragments and, consequently, an increase in local oxygen tension, creating a favorable environment for the differentiation of progenitor cells into osteoblasts. The application of allogeneic AD-MSC promotes the release of vascular endothelial growth factor (VEGF), optimizing this process, which is often impaired in cases of atrophic nonunions^8^^,^ 17.

Previous study has demonstrated high differentiation ability of canine AD-MSC into osteogenic lineage when compared to other donor sources, such as bone marrow and umbilical cord. Despite of the fact the ideal concentration of AD-MSC has not been well defined, a 1 × 10^6^ was used and early formation of bone callus was observed. Kang *et al*.^10^ used similar concentration of AD-MSC to evaluate bone healing of critical gaps in the radio of dogs with good results.

At 15 days postoperatively, 60% of cases had moderate to intense periosteal reaction, with organized formation of bone callus at the fracture site and 30% had clinical union of the fracture in the group 1. The median time of clinical radiographic union was 28.5 days (minimum of 15 and maximum of 60 days). In addition to the rapidity of the bone repair process, intense bone callus formation was also observed at 15 days in 12 cases. In contrast, the control group showed longer consolidation time with an average of 70.3 days (minimum of 15 and maximum of 120 days). It is noteworthy to observe that even with the average mean age close to the two groups, the bone healing time observed from the postoperative radiography was twice as long in the treated group as in the control group.

Clinical union can be defined as the presence of bridge callus or callus with 50% of the tibial diameter at the fracture site in at least three of the four cortices in the orthogonal projection^6^. Guiot and Déjardin^6^ demonstrated a mean radiographic bone healing time < 30 days in puppies and 42 days in adult dogs using the MIPO fixation technique in tibial fractures. Those results are in contrast with those in this study, in which puppies and adult dogs had bone consolidation visible radiographically at 15 and 30 days, respectively. Another study also reported longer times to bone healing, where puppies had an average of 30 days to bone healing^4^. It is believed that the addition of AD-MSC cell therapy to the MIPO, preserves an appropriate biological environment at the fracture site for cell signaling, neoangiogenesis stimulation and osteogenesis. Filgueira *et al.* 18 used bone marrow graft and platelet-rich plasma injected percutaneously at the fracture site in association with the MIPO technique. In the above-mentioned study, there were no cases of rejection, inflammation or complications related to percutaneous application, showing this to be a simple, effective and safe route of cell delivery. Adipose-derived mesenchymal stem cells therapy has significant advantages even when compared to studies that used common grafts. The autogenous bone marrow graft is considered the gold standard, given its compatibility, good functionality and the nontransmission of diseases. However, collection of bone tissue can prolong surgical time and result in future complications, including intermittent pain^7^. There are several ways to apply MSCs, including local application, which offers advantages by reducing the migration of cells to other organs and decreasing the host’s inflammatory response, thus increasing the effectiveness of cell therapy^19^. In addition, the percutaneous application of allogeneic AD-MSC is a noninvasive technique, as it does not require preparation and surgical access to another donor site, reducing morbidity and surgical time.

Three animals had major complications in the present study. Of these, two were the incursion of the most proximal screw end into the stifle joint space. In this case, a second surgical intervention in the immediate postoperative period was required to remove or replace the screw for a shorter one. The other major complication was the unacceptable apposition in one patient which was detected on immediate postoperative radiographs. During surgery, this patient had a hypotensive anesthetic complication, so it was decided not to intervene surgically to correct the misalignment. Despite the angular deviations generated by the failure of closed reduction in this case, the bone repair process progressed satisfactorily, and between 90 and 120 days postoperatively the bone remodeling process resulted in partial correction of these deviations.

Clinical studies evaluating the treatment of fractures have several limitations. The variability of population characteristics and factors that influence the bone repair process are difficult to control. However, prospective studies with adequate population size should mitigate these limitations and provide more reliable results.

## Conclusion

The rapid process of bone repair and the low rate of complications observed in this study suggests that the use of allogeneic AD-MSC cell therapy and MIPO is a safe, viable and effective technique for promoting bone healing in nonarticular tibial fractures in dogs.
